# Characterization of Zika Virus Endocytic Pathways in Human Glioblastoma Cells

**DOI:** 10.3389/fmicb.2020.00242

**Published:** 2020-03-06

**Authors:** Mei Li, Di Zhang, Chuntian Li, Zifeng Zheng, Ming Fu, Fengfeng Ni, Yalan Liu, Tao Du, Hanzhong Wang, George E. Griffin, Mudan Zhang, Qinxue Hu

**Affiliations:** ^1^State Key Laboratory of Virology, Wuhan Institute of Virology, Center for Biosafety Mega-Science, Chinese Academy of Sciences, Wuhan, China; ^2^University of Chinese Academy of Sciences, Beijing, China; ^3^CAS Key Laboratory of Special Pathogens and Biosafety, Wuhan Institute of Virology, Chinese Academy of Sciences, Wuhan, China; ^4^Institute for Infection and Immunity, St George’s, University of London, London, United Kingdom

**Keywords:** Zika virus, endocytosis, clathrin, cholesterol, Rab protein

## Abstract

Zika virus (ZIKV) infections can cause microcephaly and neurological disorders. However, the early infection events of ZIKV in neural cells remain to be characterized. Here, by using a combination of pharmacological and molecular approaches and the human glioblastoma cell T98G as a model, we first observed that ZIKV infection was inhibited by chloroquine and NH_4_Cl, indicating a requirement of low intracellular pH. We further showed that dynamin is required as the ZIKV entry was affected by the specific inhibitor dynasore, small interfering RNA (siRNA) knockdown of dynamin, or by expressing the dominant-negative K44A mutant. Moreover, the ZIKV entry was significantly inhibited by chlorpromazine, pitstop2, or siRNA knockdown of clathrin heavy chain, indicating an involvement of clathrin-mediated endocytosis. In addition, genistein treatment, siRNA knockdown of caveolin-1, or overexpression of a dominant-negative caveolin mutant impacted the ZIKV entry, with ZIKV particles being observed to colocalize with caveolin-1, implying that caveola endocytosis can also be involved. Furthermore, we found that the endocytosis of ZIKV is dependent on membrane cholesterol, microtubules, and actin cytoskeleton. Importantly, ZIKV infection was inhibited by silencing of Rab5 and Rab7, while confocal microscopy showed that ZIKV particles localized in Rab5- and Rab7-postive endosomes. These results indicated that, after internalization, ZIKV likely moves to Rab5-positive early endosome and Rab7-positive late endosomes before delivering its RNA into the cytoplasm. Taken together, our study, for the first time, described the early infection events of ZIKV in human glioblastoma cell T98G.

## Introduction

Zika virus (ZIKV) is a mosquito-borne virus belonging to the *Flavivirus* genus, which includes other pathogens such as dengue virus (DENV), Japanese encephalitis virus (JEV), West Nile virus (WNV), and yellow fever virus (YFV). ZIKV was originally isolated from a sentinel monkey in the Zika Forest of Uganda (Dick et al., 1952), and the first human infections were reported in 1977 in Central Java, Indonesia (Olson et al., 1981). The outbreak of ZIKV in French Polynesia and in Brazil, which expanded rapidly throughout South and Central America, raised a global health emergency (Avšic Županc and Petrovec, 2016; Bharucha and Breuer, 2016; Plourde and Bloch, 2016). Reports have now revealed the capability of ZIKV to cross the human placental barrier and, consequently, to infect the developing central nervous system (CNS) (Calvet et al., 2016). ZIKV infection in unborn fetuses showed cerebral calcifications, microcephaly, and other congenital malformations (Brasil et al., 2016; Rasmussen et al., 2016). In adults, neurological manifestation is known as Guillain–Barre syndrome, with symptoms of neuropathy and paralysis (Acosta et al., 2009; Oehler et al., 2014; Cao-Lormeau et al., 2016).

Zika virus is an enveloped, positive-sense stranded RNA virus. The nearly 11-kb open reading frame encodes three structural proteins [capsid (C) protein, precursor membrane (prM) protein, and envelope (E) protein] and seven non-structural (NS) proteins (NS1, NS2A, NS2B, NS3, NS4A, NS4B, and NS5) (Shi and Gao, 2017). The genomic RNA of ZIKV is organized within multiple copies of the protein C, forming a nucleocapsid surrounded by a host-derived lipid bilayer which contains a viral membrane protein (prM/M) and an envelope protein (E). ZIKV protein E is the major structural protein exposed on the cell surface of the particle and has been suggested to be engaged in viral attachment, penetration, and membrane fusion (Stadler et al., 1997). After internalization, flaviviruses are thought to traffic to an endosomal compartment, where a low pH induces conformational changes for viral uncoating (Gollins and Porterfield, 1986). It is known that viruses can utilize several endocytic pathways to enter host cells, including, but not limited to, clathrin-mediated endocytosis (CME), caveola/cholesterol-dependent uptake, and clathrin- and caveola-independent endocytosis such as macropinocytosis (Schelhaas, 2010). CME is the well-characterized and most common endocytic pathway employed by viruses. Some of these pathways involve dynamin II, as indicated by the beads around the neck of the endocytic indentations (Marsh and Helenius, 2006; Mercer et al., 2010). Recent studies have shown that these pathways differ from each other, and certain endocytic components can participate in more than just one pathway (Mayor and Pagano, 2007; Sandvig et al., 2008; Zhu et al., 2012). To date, most of the researches carried out on flavivirus endocytosis have been done with DENV and JEV. For instance, for productive infection of Vero and BHK-21 cells (Nawa et al., 2003; Liu et al., 2017), the functional entry of JEV is clathrin-mediated endocytosis, whereas in Neuro2a cells, its entry is clathrin-independent endocytosis (Kalia et al., 2013). DENV-1 infects Vero cells through a classical clathrin-mediated, dynamin-dependent endocytosis, while DENV-2 infects the same cell lines *via* a non-classical endocytic pathway independent of clathrin and caveolin-1, but dependent on dynamin (Acosta et al., 2009). It is probable that viruses of diverse strains may use different mechanisms to enter the same cell lines. In light of these findings, the entry of flaviviruses into different cell lines appears to be very complex and likely involves different endocytic pathways.

The cell biology of ZIKV entry remains relatively unexplored. Some reports showed that ZIKV enters Axl-expressing cells by clathrin-mediated endocytosis and traffics through early endosomes (Nowakowski et al., 2016). Axl is expressed in glial cells in the developing brain and identified as an entry factor for ZIKV infection. It was reported that chloroquine, a 4-aminoquinoline, a weak base that is rapidly imported into acidic vesicles, consequently increasing the pH, interfered with ZIKV infection in Vero cells. Furthermore, researches of ZIKV endocytosis have broadened our understanding of ZIKV intracellular trafficking in clathrin-mediated endocytosis. ZIKV fusion occurs in late endosomes (Owczarek et al., 2019). It has been increasingly appreciated that many viruses can utilize more than one entryway to infect cells (Kalia et al., 2013; Yang et al., 2013). There have been studies showing that viruses can use clathrin-independent endocytosis for productive entry and infection. It has been shown that the cell types including radial glia cells, astrocytes, endothelial cells, and microglia may be particularly vulnerable to ZIKV infection (Nowakowski et al., 2016). In the current study, we addressed the role of different endocytic molecules and pathways involved in the ZIKV infection of T98G cells. Using a combination of pharmacological and molecular approaches, we have demonstrated that both clathrin-dependent and clathrin-independent pathways can be involved in the ZIKV infection of glioblastoma T98G cells. Dynamin II, a caveolin-1-dependent membrane cholesterol, and a dynamic actin cytoskeleton are required for the ZIKV infection of T98G cells, whereas the ZIKV entry into T98G cells is independent of micropinocytosis. In addition, immunofluorescence analysis of viral colocalization with endocytic markers showed that ZIKV is trafficked to Rab5-postive early endosome and Rab7-postive later endosomes.

## Materials and Methods

### Cells and Viruses

The African green monkey kidney cell line Vero (ATCC) and the human glioblastoma cell line T98G (ATCC) were cultured in minimum essential medium (MEM; Gibco) containing 10% fetal bovine serum (FBS; Gibco) and 100 U/ml penicillin/streptomycin. Baby hamster kidney BHK-21 cells (ATCC) were maintained in Dulbecco’s modified Eagle’s medium (DMEM; HyClone) supplemented with 10% FBS (Gibco) and 100 U/ml penicillin/streptomycin. All cell lines were grown at 37°C in the presence of 5% CO_2_. The ZIKV strain (Zika virus/s201/2016/China, GenBank: kv963796) was propagated in Vero cells utilizing MEM containing 2% FBS. The JEV strain SA14-14 was propagated in BHK-21 cells grown in DMEM containing 2% FBS. Pseudotyped vesicular stomatitis virus (VSVpv) bearing the VSV-G envelope protein was generated as described previously (Fukuhara et al., 2011).

### Plaque Assay

Zika virus titer was determined by plaque assay using Vero cells. Viral stocks were 10-fold serially diluted in MEM three times. For each dilution, a 500 μl sample was added to one well in a 12-well plate containing Vero cells at about 100% confluency. The infected cells were incubated at 37°C for 1 h and rocked back and forth gently every 15 min. After the incubation, the dilution was removed and 1 ml of a methyl cellulose overlay containing 2% FBS and 1% penicillin/streptomycin was added to each well, and the plate was incubated at 37°C for 5 days. Following the incubation, the methyl cellulose overlay was removed, and the plate was stained with 1% crystal violet containing 4% formaldehyde for 15 min. Visible plaques were counted, and viral titers were counted as plaque-forming units per milliliter (PFU/ml). The JEV titer was determined by plaque assay using BHK-21 cells, as described above.

### Cell Infection and Drug Treatment

To test ZIKV infection in the presence of various drugs, T98G cells were seeded into 12-well plates for 24 h until they were completely confluent and then pretreated with the indicated concentrations of chloroquine (Sigma), NH_4_Cl (Sigma), chlorpromazine (Sigma), pitstop2 (Sigma), dynasore (Abcam), Filipin III (MCE, NJ, United States), methyl-β-cyclodextrin (MβCD) (Sigma), Jasplakinolide (Abcam), nocodazole (Sigma), 5-(*N*-ethyl-*N*-isopropyl)amiloride (EIPA) (MCE, NJ, United States), or wortmannin (MCE, NJ, United States) before or during viral infection. Cell viability following different drug treatments was evaluated by a cell viability assay, as described below. After treatment, ZIKV was added into each well. Total RNA was extracted by using NucleoSpin RNA Plus (MN-740984), and the viral RNA level was quantitated by using a reverse transcription quantitative real-time PCR (RT-qPCR) assay, as described previously (Xu et al., 2016). Data are presented as 2^–ΔΔ^*^*CT*^* values from quadruplicate samples.

### Cell Viability Assay

The 3-(4,5-dimethylthiazol-2-yl)-2,5-diphenyl tetrazolium bromide (MTT) assay (Sigma) was used to determine cell viability upon treatment with drugs. In brief, T98G cells were treated with the drugs for 1 h and subsequently cultured in medium containing 2% FBS. After cultivation at 37°C for 24 h, MTT (5 mg/ml in phosphate-buffered saline, PBS) was added and the cells were further cultured for 4 h. The supernatants were then removed from the wells, and MTT formazan was dissolved following the addition of 50 μl dimethyl sulfoxide (DMSO) per well. The plates were subjected to absorbance reading using an ELISA plate reader (BioTek) at a wavelength of 490 nm.

### RNA-Mediated Interference

The following small interfering RNA (siRNA) oligonucleotides were synthesized by GenePharma: AUCCGCGCGAUAGUACGUATT for negative control, UCCAAUUCGAAGACCAAUUTT for clathrin heavy chain (CHC), and AGCCGAGCUGAGCGAGAAGCA for caveolin-1 (Masuyama et al., 2009). The siRNAs targeting vacuolar ATPase (VTPase, sc-42686), dynamin (sc-43736), Rab5 (sc-36344), and Rab7 (sc-29460), respectively, were purchased from Santa Cruz. T98G cells were transfected with the indicated siRNAs using a HiPerFect Transfection Reagent (Qiagen) according to the manufacturer’s protocol (Zheng et al., 2018). Knockdown efficiency was measured by Western blot. The function of siRNA targeting CHC and dynamin II was confirmed by the uptake of Alexa Fluor 555-conjugated transferrin (10 μg/ml, Molecular Probes).

### Transfection of T98G Cells

Plasmids expressing green fluorescent protein (GFP)-tagged wild-type (WT) or dominant-negative (DN; K44A) dynamin and constructs expressing wild-type caveolin-1 (GFP-cav-1 WT) or dominant-negative caveolin-1 (GFP-cav-1 DN) mutants were kindly provided by Dr. Yong-zhe Zhu (Second Military Medical University, Shanghai). Plasmids pEGFP-Rab5 and pEGFP-Rab7 were kindly provided by Dr. Zongqiang Cui (Li Q. et al., 2017). T98G cells were seeded onto 35-mm glass-bottom culture dishes and grown overnight until 80% confluence. Of the plasmid, 2 μg was mixed with 200 μl of Opti-MEM containing 4 μl of X-tremeGENE HP DNA Transfection Reagent (Roche). The transfection reagent:DNA complex was incubated for 30 min at room temperature. Following a 24 h transfection, the cells were infected with ZIKV at a multiplicity of infection (MOI) of 0.5.

### Quantification of ZIKV Infection by Flow Cytometry

The ZIKV infection of cells transfected with plasmids was measured by flow cytometry. Cells transfected with the indicated plasmids were infected with ZIKV at an MOI of 0.5. After incubation, the infected cells were washed off with chilled PBS and fed with fresh MEM. At 24 h post-infection, the cells were treated with a BD Cytofix/CytopermTM Fixation/Permeabilization Kit (cat. 554714, Becton Dickinson) and incubated with an antibody against ZIKV envelope protein (4G2, Merck-Millipore) for 1 h on ice, followed by Alexa Fluor 555-labeled donkey anti-mouse IgG (H + L, A0460, Beyotime). The cells were washed three times with a flow cytometry buffer and fixed with 1% paraformaldehyde. At least 5,000 events were collected for each sample and analyzed using a Becton Dickinson FACSCantoII flow cytometer.

### Western Blotting

Cells were lysed in radioimmunoprecipitation assay (RIPA) buffer and the cell lysates were separated by sodium dodecyl sulfate–polyacrylamide gel electrophoresis (SDS-PAGE), and then transferred onto polyvinylidene fluoride (PVDF) membranes. The membranes were incubated in PBS with non-fat milk and probed with an anti-CHC antibody (1:1,000; ab21679, Abcam), anti-dynamin II antibody (1:1,000; ab151555, Abcam), anti-caveolin-1 antibody (1:1,000; C4490, Sigma), anti-Rab5 antibody (1:1,000; ab18211, Abcam), or an anti-Rab7 antibody (1:1,000; ab126712, Abcam) for 1 h at room temperature and subsequently washed three times with 0.1% Tween 20/PBS, followed by an incubation for 1 h with horseradish peroxidase (HRP)-conjugated goat anti-rabbit secondary antibody (1:10,000; BA1054, Boster) or HRP-conjugated goat anti-mouse secondary antibody (1:10,000; BA1050, Boster). The membranes were washed three times with 0.1% Tween 20/PBS and visualized by exposure to FluorChem HD2 Imaging System (Alpha Innotech) after the addition of a chemiluminescent substrate (SuperSignal West Dura Extended Duration Substrate; 34075; Thermo Scientific Pierce) (Li M. et al., 2017).

### Immunofluorescence

T98G cells transfected with plasmids or siRNA were infected with ZIKV at an MOI of 0.5 and incubated for 1 h at 37°C. At 24 h post-infection, cells on 35-mm glass-bottom culture dishes were washed three times with PBS, followed by the addition of 4% (*w*/*v*) cold paraformaldehyde. The cells were fixed for 10 min at room temperature, washed three times with PBS for 5 min, and then blocked with a blocking buffer (5% FBS and 0.3% Triton X-100 in PBS) for 1 h. The cells were incubated overnight at 4°C with the anti-ZIKV E antibody at a dilution of 1:200, followed by incubation with an Alexa Fluor 555-labeled donkey anti-mouse IgG (H + L, A0460, Beyotime) or Alexa Fluor 488-labeled goat anti-mouse IgG (H + L, A-11001, Thermo Fisher) at a dilution of 1:500 in PBS–3% (*w*/*v*) bovine serum albumin (BSA) for 1 h at room temperature in the dark. Nuclei were dyed with 4′,6-diamidino-2-phenylindole (DAPI; C1006, Beyotime). Stained cells were analyzed under an Olympus IX73 microscope using a × 20 objective or by confocal microscopy (Andor Dragonfly 202 or Nikon A1 MP) using a × 60 oil objective with a 1.5-fold optical zoom.

### Assay of Viral Penetration

T98G cells were plated in a six-well plate and incubated with ZIKV at an MOI of 10 for 1 h at 4°C. The cells were washed three times with cold PBS, followed by the addition of MEM containing 2% FBS. After incubation at 37°C for 1 h, the cells were washed with cold PBS and treated with proteinase K (1 mg/ml) for 45 min at 4°C to remove the absorbed but not yet internalized virus. After the proteinase K was inactivated by treatment with 2 mM phenylmethylsulfonyl fluoride (PMSF) in cold PBS containing 3% BSA, the cells were further washed with PBS–0.2% BSA. The samples were then immediately processed for total RNA extraction. Total RNA was extracted using NucleoSpin RNA Plus (MN-740984) and quantitated by a RT-qPCR assay, as described previously (Xu et al., 2016). Data are presented as 2^–ΔΔ^*^*CT*^* values from quadruplicate samples.

### Infectious Center Assay

The internalization of ZIKV was measured by an infectious center assay (Wang et al., 2016). T98G cells were treated with the indicated inhibitor for 1 h and then incubated with ZIKV at an MOI of 10 at 4°C for 1 h. The cells were then moved to 37°C in the presence or absence of the inhibitors. After 1 h of incubation, the cells were treated with proteinase K (1 mg/ml) at 4°C for 45 min, as mentioned above. Finally, cell pellets were resuspended in MEM, and 10-fold serial dilutions of the cell suspensions were plated onto Vero monolayers and overlaid with MEM containing 1% methyl cellulose and 2% FBS. The cells were then incubated at 37°C for 5 days, fixed in 4% formaldehyde, and stained with 1% crystal violet.

### Luciferase Assay

Briefly, 1 day prior to infection, 4 × 10^4^ T98G cells per well were seeded in a 96-well plate in MEM containing 10% FBS, penicillin, and streptomycin (both at 100 U/ml) and incubated at 37°C with 5% CO_2_ for 48 h. Fifty TCID_50_ pseudotyped VSV were added into the pre-seeded T98G cells. After 48 h, the medium was removed and the cells were washed and lysed. Luciferase activity was measured using a luciferase assay kit, according the manufacturer’s instructions (Promega). All conditions were done in duplicate. Background luciferase activity was subtracted with the luciferase activity from uninfected cells.

### Statistical Analysis

Statistical analyses and calculations were performed with GraphPad Prism 7 software. Student’s *t* test was used for a comparison between the untreated and treated groups. Statistical significance is depicted in the figures: ^∗^*P* < 0.05, ^∗∗^*P* < 0.01, ^∗∗∗^*P* < 0.001. A *P* value less than 0.05 was considered as statistically significant.

## Results

### ZIKV Infection Is Dependent on Acidic pH

**FIGURE 1:**
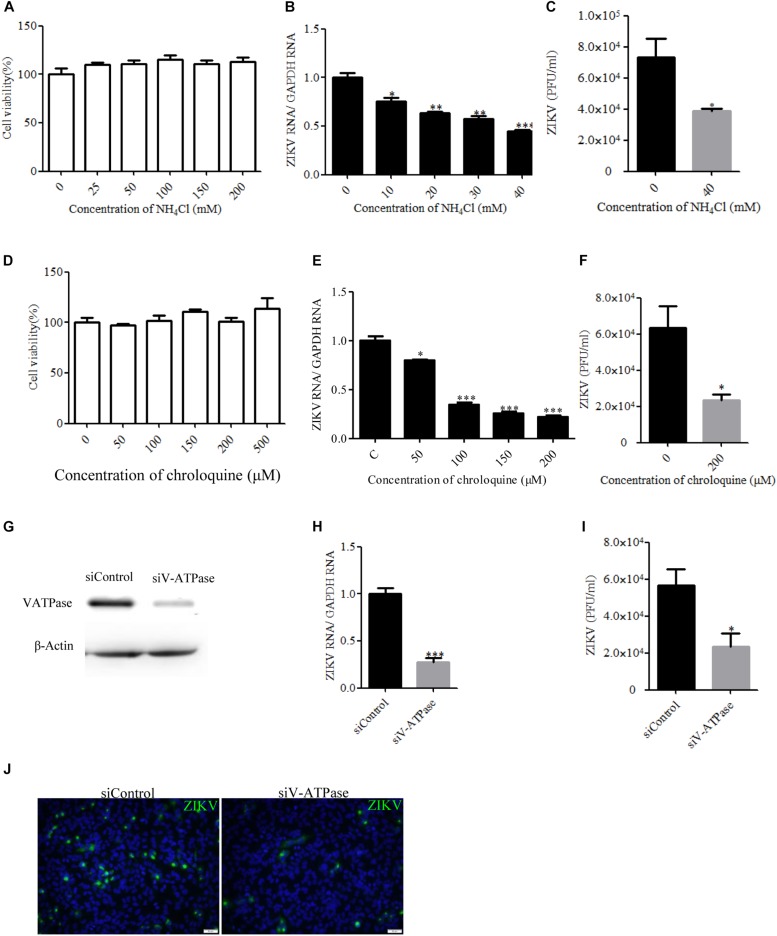
Zika virus (ZIKV) infection requires acidic endosomal pH. **(A**,**D)** Cells treated with increasing concentrations of NH_4_Cl or chloroquine were assessed by the MTT assay to test cell viability. **(B**,**E)** Treatment with NH_4_Cl or chloroquine inhibited ZIKV infection. T98G cells treated with NH_4_Cl **(B)** or chloroquine **(E)** were incubated with ZIKV for 1 h at 37°C in the presence of drugs. At 24 h post-infection, the infected cells were lysed to determine viral RNA copy numbers by RT-qPCR. **(C**,**F)** Treatment with NH_4_Cl or chloroquine inhibited ZIKV infection. T98G cells treated with NH_4_Cl **(C)** or chloroquine **(F)** were incubated with ZIKV for 1 h at 37°C. At 48 h post-infection, the titers of supernatant viruses were determined by plaque assay. **(G)** To verify V-ATPase knockdown, protein samples from cells expressing each siRNA construct were analyzed by Western blotting. **(H**,**I)** T98G cells were transfected with siRNA targeting V-ATPase or control siRNA for 48 h and then infected with ZIKV at an MOI of 0.5. At 24 h post-infection, the cells were lysed to determine viral RNA copy numbers **(H)**. At 48 h post-infection, the titers of supernatant viruses were determined by plaque assay **(I)**. **(J)** At the same time, the cells were fixed with 4% paraformaldehyde, incubated with an anti-ZIKV E antibody (*green*), and visualized by an Olympus microscope. Nuclei were counterstained with DAPI (*blue*). *Scale bars* in all panels represent 50 μm. One representative experiment out of three is shown **(G**,**J)**. The data shown are the mean ± SD of three independent experiments **(A**–**F**,**H**,**I)**. *P < 0.05; **P < 0.01; ***P < 0.001.

Endocytic entry into host cells by most enveloped viruses requires a low-pH step to trigger fusion of the viral envelope with the endosome membrane, which leads to the release of the nucleocapsid into the cytosol. To address the role of acidic pH in the ZIKV infection of T98G cells, the effect of ammonium chloride (NH_4_Cl) and chroloquine on ZIKV infection was investigated. We used a ZIKV strain (Zika virus/s201/2016/China, GenBank: kv963796) in which neurotropism has been well characterized (Deng et al., 2016). NH_4_Cl is a lysosomotropic weak base that raises the pH of intracellular acidic vesicles (Zhu et al., 2012), while chroloquine is an inhibitor of endosomal acidification (Silva et al., 2007). We first tested the drugs’ cytotoxic effects using the MTT cell viability assay. NH_4_Cl did not impact cell viability at concentrations of 40 mM or lower, whereas the working concentrations of chroloquine range from 0 to 200 μM (Figures 1A,D). Following the treatment of T98G cells with increasing concentrations of NH_4_Cl or chroloquine, copies of the viral genomic RNAs of progeny viruses were significantly decreased in a dose-dependent fashion (Figures 1B,E). Plaque assays showed that there was an apparently reduced production of infectious virions in the presence of drugs (Figures 1C,F). Subsequently, we examined the low pH requirement for ZIKV infection by silencing the expression of vacuolar ATPase (V-ATPase), a proton pump, which is key to the establishment of low pH in endosomal compartments. The knockdown efficiency of V-ATPase siRNA was analyzed by Western blot (Figure 1G). As shown in Figures 1H–J, siV-ATPase (siRNA targeting V-ATPase) treatment of cells significantly reduced the copies of viral genomic RNAs and the production of infectious virions. Taken together, these data demonstrate that ZIKV infects T98G cells in a pH-dependent manner.

### ZIKV Endocytosis Into T98G Cells Depends on Dynamin

**FIGURE 2:**
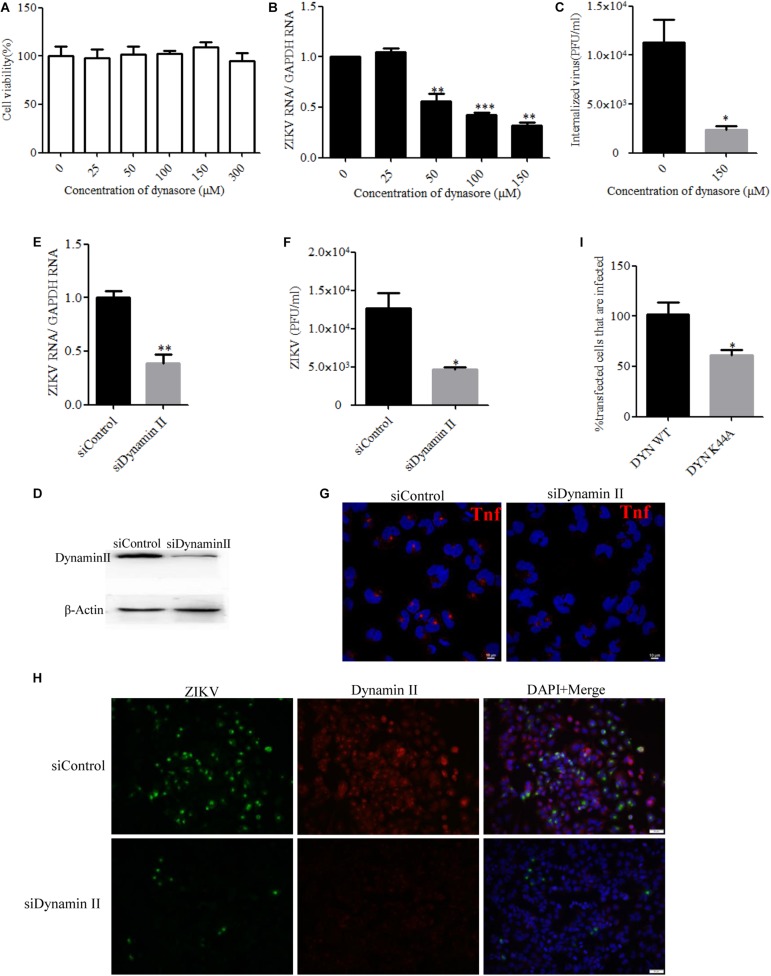
Zika virus (ZIKV) entry depends on dynamin II. **(A)** Cell viability upon dynasore treatment was assessed by the MTT assay. **(B**,**C)** Treatment with dynasore inhibited the ZIKV entry into T98G cells. T98G cells treated with dynasore were incubated with ZIKV at a multiplicity of infection (MOI) of 10 for 1 h at 4°C and then shifted to 37°C for 1 h. The internalized viruses were determined by RT-qPCR **(B)** and infectious center assay **(C)**. **(D)** To verify dynamin II knockdown, protein samples from the cells transfected with each siRNA were analyzed by immunoblotting for dynamin II. **(E**,**F)** T98G cells were transfected with siRNA targeting dynamin II or control siRNA for 48 h, followed by infection with ZIKV at an MOI of 10 for 1 h at 4°C and then shifted to 37°C for 1 h. The internalized viruses were determined by RT-qPCR **(E)** and infectious center assay **(F)**. **(G)** T98G cells were transfected with siRNA targeting dynamin II or control siRNA for 48 h and then incubated with 10 μg/ml AF-555-labeled transferrin for 10 min at 37°C. The cells were fixed and stained with DAPI. *Scale bars* in all panels represent 10 μm. **(H)** T98G cells were transfected with siRNA targeting dynamin II or control siRNA for 48 h, followed by infection with ZIKV at an MOI of 0.5. At 48 h post-infection, the cells were fixed and analyzed under an Olympus microscope. *Scale bars* in all panels represent 50 μm. **(I)** The cells transfected with the dynamin II wild-type or dominant-negative plasmid construct were infected with ZIKV at an MOI of 0.5. At 48 h post-infection, the cells were analyzed by flow cytometry. One representative experiment out of three is shown **(D)**. Representative confocal images from three independent experiments are shown **(G**,**H)**. The data shown are the mean ± SD of three independent experiments **(A**–**C**,**E**,**F**,**I)**. *P < 0.05; **P < 0.01; ***P < 0.001.

Dynamin II, a GTPase, is the most studied membrane fusion machinery and plays a critical role in pinching off vesicles in clathrin- and caveola-dependent endocytosis (Marsh and Helenius, 2006). To investigate whether dynamin II is involved in the ZIKV entry into T98G cells, the effect of dynasore, a cell-permeable, non-competitive inhibitor of dynamin GTPase activity, was evaluated (Macia et al., 2006). No apparent cytotoxicity was observed when the cells were treated with dynasore up to 150 μM (Figure 2A). We observed that dynasore inhibited ZIKV entry in a dose-dependent manner (Figures 2B,C). To further determine the role of dynamin II during ZIKV entry, we silenced dynamin II by siRNA treatment. Western blotting showed a reduced expression of dynamin II by siRNA compared to the control siRNA (Figure 2D). T98G cells were transfected with a specific siRNA against dynamin II or a control siRNA and then infected with ZIKV at an MOI of 10. Depletion of dynamin II significantly reduced ZIKV internalization in T98G cells (Figures 2E,F). A significant decrease of the mean fluorescence intensity (MFI) of transferrin was observed in the cells treated with the specific siRNA against dynamin II (Figure 2G). When siDynamin-transfected cells were infected with ZIKV and examined by immunofluorescence, we observed a significant reduction in the number of ZIKV-infected cells compared to that with the control siRNA (siControl) treatment (Figure 2H). To further confirm the role of dynamin in the ZIKV infection of T98G cells, the GFP-tagged versions of the wild-type form of dynamin II (DYN WT) and the dominant-negative mutant GFP-dyn II K44A (DYN K44A) were used (Damke et al., 1994; Oh et al., 1998). T98G cells were transfected with both constructs, and after 48 h of transfection, the cells were infected with ZIKV (MOI = 0.5). The expression of the DN dynamin II K44A mutant resulted in an approximately 35% inhibition of the number of ZIKV E-expression cells (Figure 2I). Collectively, it is reasonable to conclude that the entry of ZIKV into T98G cells is dependent on the functionality of dynamin II.

### ZIKV Entry Into T98G Cells Is Clathrin-Dependent

**FIGURE 3:**
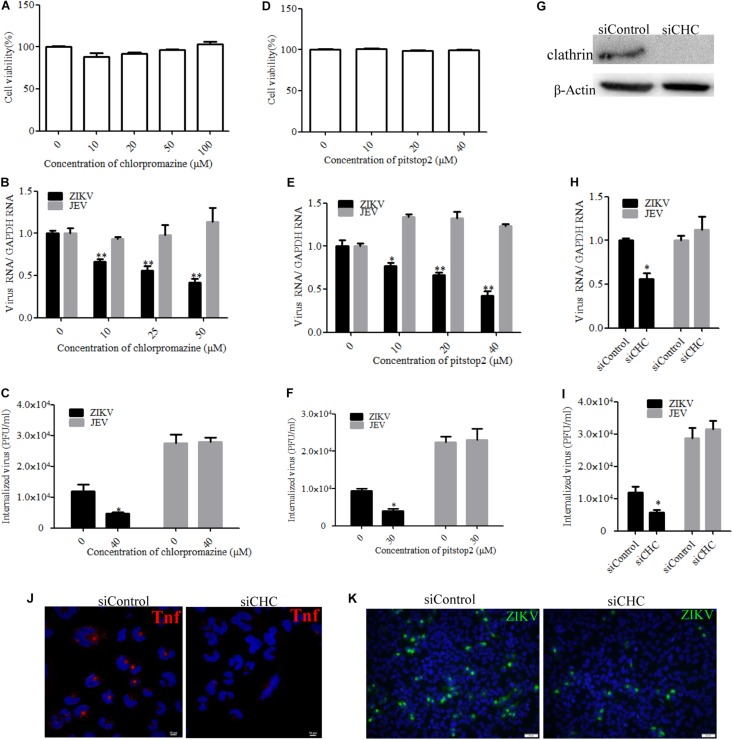
Clathrin is required for the Zika virus (ZIKV) infection of T98G cells. **(A**,**D)** Cell viability upon chlorpromazine treatment was assessed by the MTT assay. **(B**,**C)** Treatment with chlorpromazine inhibited the ZIKV entry into T98G cells. T98G cells treated with chlorpromazine were incubated with ZIKV or Japanese encephalitis virus (JEV) at a multiplicity of infection (MOI) of 10 for 1 h at 4°C and then shifted to 37°C for 1 h. The internalized viruses were determined by RT-qPCR **(B)** and infectious center assay **(C)**. **(E**,**F)** Treatment with pitstop2 inhibited the ZIKV entry into T98G cells. T98G cells treated with increasing concentrations of pitstop2 were infected with ZIKV or JEV at an MOI of 10 for 1 h at 4°C and then shifted to 37°C for 1 h. The internalized viruses were determined by RT-qPCR **(E)** and infectious center assay **(F)**. **(G)** To verify the knockdown of clathrin heavy chain, protein samples from the cells transfected with each siRNA were analyzed by immunoblotting for clathrin heavy chain. **(H**,**I)** T98G cells were transfected with siRNA targeting clathrin heavy chain or control siRNA for 48 h, followed by infection with ZIKV or JEV at an MOI of 10 for 1 h at 4°C and then shifted to 37°C for 1 h. The internalized viruses were determined by RT-qPCR **(H)** and infectious center assay **(I)**. **(J)** T98G cells were transfected with siRNA targeting clathrin heavy chain or control siRNA for 48 h and then incubated with 10 μg/ml AF-555-labeled transferrin for 10 min at 37°C. The cells were fixed and stained with DAPI (*blue*). *Scale bars* in all panels represent 10 μm. **(K)** T98G cells were transfected with siRNA targeting clathrin heavy chain or control siRNA for 48 h, followed by infection with ZIKV at an MOI of 0.5. At 48 h post-infection, the cells were fixed and analyzed by an Olympus microscope. Nuclei were counterstained with DAPI (*blue*). *Scale bars* in all panels represent 50 μm. One representative experiment out of three is shown **(G)**. Representative confocal images from three independent experiments are shown **(J**,**K)**. The data shown are the mean ± SD of three independent experiments **(A**–**C**,**E**,**F**,**H**,**I)**. *P < 0.05; **P < 0.01.

Zika virus has been shown to exploit the clathrin-dependent pathway to enter CHME3 and HT1080 cells (Persaud et al., 2018). Therefore, the role of clathrin in the ZIKV entry into T98G cells was tested by employing different strategies to disrupt the pathway. First, chlorpromazine and pitstop2 were used to inhibit clathrin-mediated endocytosis. Chlorpromazine prevents the assembly of clathrin-coated pits at the cell surface and induces the assembly of a clathrin lattice on endosomes (Wang et al., 1993). Pitstop2 is an inhibitor of the interaction of amphiphysin with the N-terminal domain of clathrin heavy chain (Dutta et al., 2012). No apparent cytotoxicity was observed when the cells were treated with chlorpromazine up to 50 μM and pitstop2 up to 30 μM (Figures 3A,D). We observed that chlorpromazine and pitstop2 inhibited ZIKV internalization into cells in a dose-dependent manner (Figures 3B,E), whereas the viral RNA copies of JEV, another member of the Flaviviridae, remained unchanged. The infectious center assay showed an apparently reduced ZIKV internalization in the presence of drugs. Of note is that chlorpromazine or pitstop2 treatment had no effect on JEV internalization (Figures 3C,F). Furthermore, we assessed the role of clathrin during ZIKV infection by siRNA knockdown of CHC. Depletion of CHC in T98G cells was confirmed by Western blot (Figure 3G). To assess whether CHC depletion affects ZIKV entry, T98G cells were transfected with siRNA specifically against CHC followed by infection with ZIKV at an MOI of 10, showing that the depletion of CHC led to a significant decrease of ZIKV internalization into T98G cells (Figures 3H,I). A significantly decreased MFI of transferrin was observed in the cells treated with the specific siRNA against CHC (Figure 3J). When siCHC-transfected cells were infected with ZIKV and examined by immunofluorescence, the number of ZIKV-infected cells reduced compared to that in the cells transfected with siControl (Figure 3K). These data together indicate that the ZIKV entry into T98G cells can be mediated by a clathrin-dependent pathway.

### ZIKV Enters T98G Cells *via* a Caveola-Mediated Endocytosis Pathway

**FIGURE 4:**
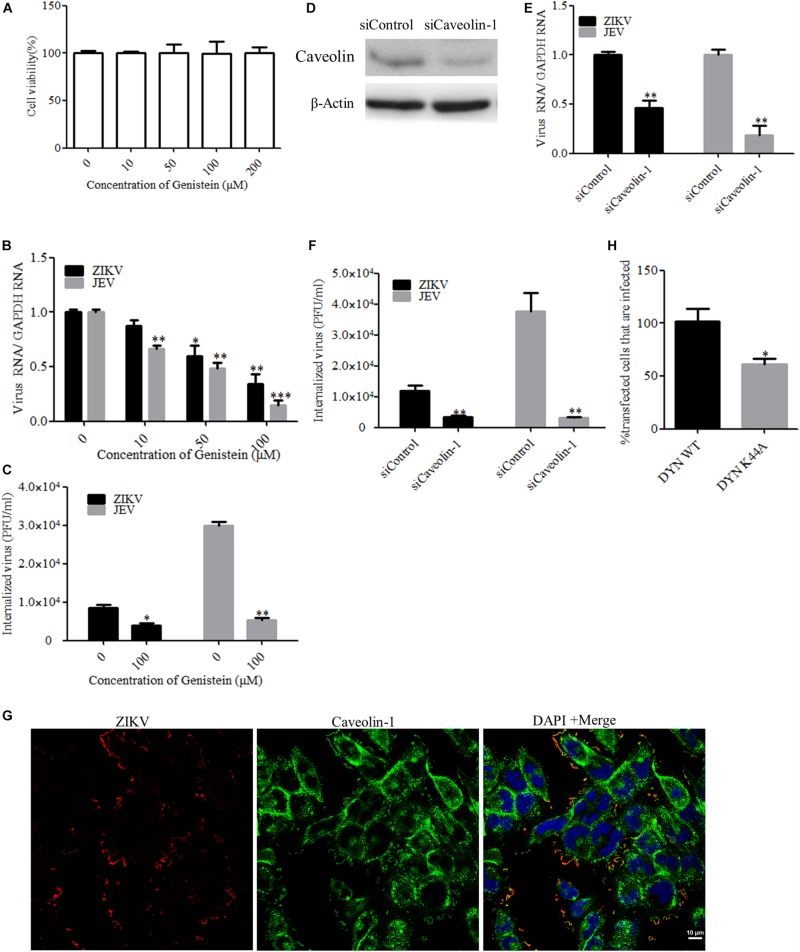
Zika virus (ZIKV) enters T98G cells *via* a caveola-mediated endocytosis pathway. **(A)** Cell viability upon genistein treatment was assessed by the MTT assay. **(B**,**C)** Treatment with genistein inhibited ZIKV entry. T98G cells were pretreated with increasing concentrations of genistein for 1 h at 37°C, followed by infection with ZIKV or Japanese encephalitis virus (JEV) at an MOI of 10, with the drug being maintained during the adsorption period of 1 h at 37°C. The internalized viruses were determined by RT-qPCR **(B)** and infectious center assay **(C)**. **(D)** To verify caveolin-1 knockdown, protein samples from the cells expressing each siRNA construct were analyzed by immunoblotting for caveolin-1. **(E**,**F)** T98G cells were transfected with siRNA targeting caveolin-1 protein or control siRNA for 48 h, followed by infection with ZIKV or JEV. The internalized viruses were determined by RT-qPCR **(E)** and infectious center assay **(F)**. **(G)** Colocalization of caveolin-1 with ZIKV during the early stage of infection. Cells grown on glass coverslips in six-well plates were infected with ZIKV at 4°C for 1 h and then shifted to 37°C for 5 min. The cells were then fixed with 4% paraformaldehyde, stained with mouse anti-ZIKV E antibody (*red)* and rabbit anti-caveolin-1 (*green*), and examined by confocal microscopy. *Scale bars* in all panels represent 10 μm. **(H)** The inhibitory effect of caveolin-1 dominant-negative construct on ZIKV infection was determined by flow cytometry. One representative experiment out of three is shown **(D)**. Representative confocal images from three independent experiments are shown **(G)**. The data shown are the mean ± SD of three independent experiments **(A**–**C**,**E**,**F**,**H)**. *P < 0.05; **P < 0.01; ***P < 0.001.

In addition to participating in clathrin-dependent endocytosis, dynamin has been implicated to be involved in several other endocytic pathways. Another form of endocytosis that requires dynamin is caveola-mediated uptake (Doherty and McMahon, 2009). Caveolae consist of caveolin membrane proteins and can bud from the plasma membrane, resulting in the formation of vesicles which are involved in virus entry. Caveolae are usually associated with lipid rafts and characterized by the presence of caveolin-1 protein. Genistein is a tyrosine kinase inhibitor and causes local disruption of the actin network at the site of endocytosis, which is known to be an indispensable event in caveola-mediated uptake. Here, we tested the effect of genistein, a specific tyrosine kinase inhibitor, on ZIKV entry. First, T98G cells were treated with increasing concentrations of genistein, followed by the MTT assay, showing that no apparent cytotoxicity was observed when the cells were treated with genistein up to 200 μM (Figure 4A). As shown in Figures 4B,C, the copies of ZIKV and JEV genomic RNAs and the internalization of viruses were decreased in the cells treated with increasing concentrations of genistein compared to those in the untreated control. It has been shown that, in the absence of caveolins, no caveolae were observed, and when caveolins were expressed in cells that lack caveolae, the formation of caveola was decreased (Fra et al., 1995). To block the caveola-mediated endocytosis, we used siRNA to silence caveolin-1 expression. The knockdown efficiency of siRNA targeting caveolin-1 (siCaveolin-1) was analyzed by Western blotting (Figure 4D). T98G cells were transfected with siCaveolin-1 or a control siRNA and then infected with ZIKV or JEV. RT-qPCR showed that there were significantly fewer ZIKV and JEV RNA copies in siCaveolin-1-transfected cells than those in siControl-transfected cells (Figure 4E), while the infectious center assay showed that ZIKV or JEV entry into T98G cells was reduced in the cells transfected with siCaveolin-1 compared to that in the cells transfected with siControl (Figure 4F). T98G cells were incubated with ZIKV at an MOI of 10 at 4°C for 1 h to allow for attachment and then shifted to 37°C for 5 min. The localization of ZIKV and caveolin-1 was analyzed by confocal microscopy. The results showed that ZIKV colocalized with caveolin-1 after 5 min incubation at 37°C (Figure 4G). In addition, T98G cells were transfected with the caveolin-1 WT and DN constructs, respectively, and then infected with ZIKV (Patel et al., 2009). Virus-infected cells were quantitated using flow cytometry. The overexpression of caveolin-1 DN results in a 58% reduction in the number of ZIKV-infected cells compared to that in the cells transfected with the caveolin-1 WT (Figure 4H). These data strongly suggest that the ZIKV infection of T98G cells is dependent on caveola-mediated endocytosis.

### Cholesterol Is Required for ZIKV Infection

**FIGURE 5:**
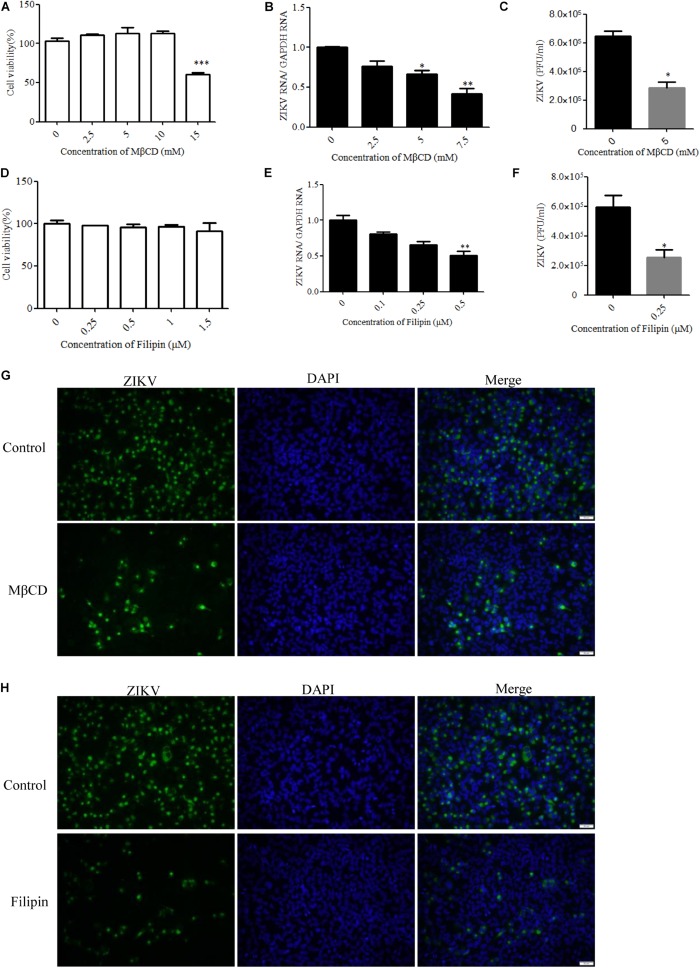
Cholesterol is required for Zika virus (ZIKV) infection. **(A**,**D)** Cell viability upon methyl-β-cyclodextrin (MβCD) and filipin treatment was assessed, as described in the text. **(B**,**E)** Treatment with MβCD or filipin inhibited ZIKV infection. T98G cells were pretreated with increasing concentrations of MβCD or filipin for 1 h at 37°C. The cells were extensively washed, followed by infection with ZIKV at a multiplicity of infection (MOI) of 5. At 24 h post-infection, the infected cells were lysed to quantitate viral RNA copy numbers by RT-qPCR. **(C**,**F)** At 48 h post-infection, the titers of supernatant viruses were determined by plaque assay. **(G)** The cells were pretreated with 7.5 mM MβCD for 1 h, extensively washed, and then infected with ZIKV at an MOI of 5. At 24 h post-infection, the cells were fixed and stained with an anti-ZIKV envelop antibody (*green*). The cells were fixed and stained with DAPI (*blue*). **(H)** The cells were pretreated with 1 μm filipin for 1 h, extensively washed, and then infected with ZIKV at an MOI of 5. At 24 h post-infection, the cells were fixed and stained with an anti-ZIKV E antibody (*green*). Nuclei were counterstained with DAPI (*blue*). *Scale bars* in all panels represent 50 μm. Representative confocal images from three independent experiments are shown **(G**,**H)**. The data shown are the mean ± SD of three independent experiments **(A**–**F)**. *P < 0.05; **P < 0.01; ***P < 0.001.

Cholesterol-dependent primary endocytic vesicle formation is one of the characteristics of the caveola-dependent pathway (Parton and Richards, 2003). Several studies have demonstrated that lipid raft and cholesterol not only play vital roles in cellular pathways but also have critical functions in virus infection (Marsh and Helenius, 2006; Lee et al., 2008). We next examined whether ZIKV infection was affected by the expression level of membrane cholesterol, which is a major determinant of many endocytic processes (Parton and Richards, 2003). Cholesterol has been suggested to be an indicator of caveola/lipid raft endocytosis, but such cholesterol dependency does not necessarily reflect a specific pathway of entry (Mayor and Pagano, 2007). For dengue and West Nile flaviviruses, cholesterol was shown to be required for infection (Lee et al., 2008; Medigeshi et al., 2008; Puerta-Guardo et al., 2010). Mouse neural stem cells depleted of cholesterol before JEV infection also demonstrated a reduction both in viral load and in the production of infective virus particles (Das et al., 2010). To determine whether ZIKV infection before trafficking to the acidic vesicles was sensitive to the perturbation of lipid rafts, we evaluated the effects of MβCD, a drug selectively extracting cholesterol from the plasma membrane (Kilsdonk et al., 1995; Acosta et al., 2009), and filipin, a compound that binds selectively to cholesterol-rich microdomains on virus infection (Bolard, 1986). Because MβCD and filipin are virucidal to ZIKV (Supplementary Figure S2), the cells were pretreated with drugs and then extensively washed prior to the addition of ZIKV. Depletion of cholesterol was conducted by treating the cells with MβCD or filipin. The cytotoxic effects of MβCD and filipin were evaluated by the MTT assay. T98G cells tolerated up to 10 mM MβCD and 1 μM filipin (Figures 5A,D). Subsequently, T98G cells were pretreated with MβCD or filipin for 1 h, extensively washed, and infected with ZIKV. RT-qPCR showed that the copies of ZIKV genomic RNAs in the treated cells were significantly reduced in a dose-dependent manner (Figures 5B,E). There was also a reduced production of infectious virions upon drug treatment (Figures 5C,F). Indirect immunofluorescence assay showed that the cells treated with 7.5 mM MβCD resulted in a significant decrease of ZIKV infection (Figure 5G). In parallel, when T98G cells were pretreated with 0.5 μM filipin, ZIKV infection was also inhibited (Figure 5H). Both treatments resulted in a remarkable inhibition of ZIKV infection, highlighting an essential role of cholesterol in the ZIKV infection of T98G cells.

### Role of Actin Cytoskeleton and Microtubules in ZIKV Infection of T98G Cells

**FIGURE 6:**
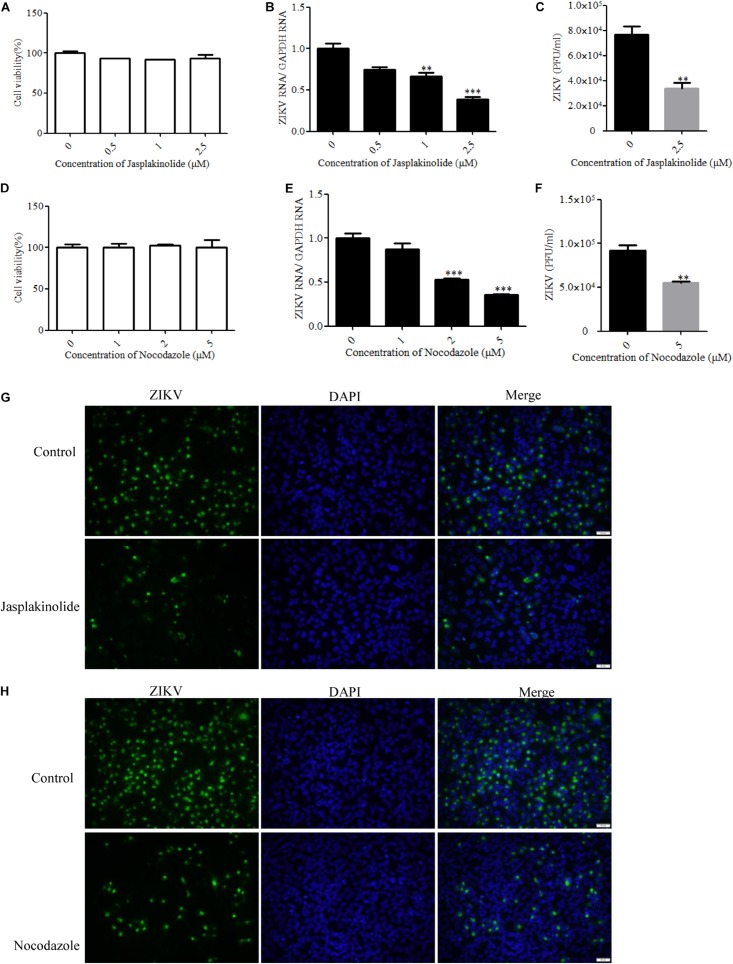
Role of microtubules and the actin cytoskeleton in Zika virus (ZIKV) infection. **(A**,**D)** Assessment of cell viability upon jasplakinolide or nocodazole treatment. **(B**,**E)** T98G cells were pretreated with jasplakinolide or nocodazole and inoculated with ZIKV at a multiplicity of infection (MOI) of 0.5. At 24 h post-infection, the cells were lysed to determine the number of viral RNA copies. **(C**,**F)** At 48 h post-infection, the titers of supernatant viruses were determined by plaque assay. **(G)** The cells were treated with 2.5 μM jasplakinolide for 1 h and then infected with ZIKV at an MOI of 5 in the presence of the inhibitor. At 24 h post-infection, the cells were fixed and stained with an anti-ZIKV envelop antibody (*green*). The cells were fixed and stained with DAPI (*blue*). **(H)** The cells were treated with 5 μm nocodazole for 1 h and then infected with ZIKV at an MOI of 5 in the presence of the inhibitor. At 24 h post-infection, the cells were fixed and stained with an anti-ZIKV envelop antibody (*green*). Nuclei were counterstained with DAPI (*blue*). Representative confocal images from three independent experiments are shown **(G**,**H)**. *Scale bars* in all panels represent 50 μm. The data shown are the mean ± SD of three independent experiments **(A**–**F)**. *P < 0.05; **P < 0.01; ***P < 0.001.

Many viruses use the cytoskeleton network of the cell as a transport system. Both actin and microtubules have been reported to be involved in the JEV infection of human neuroblastoma cells (Henry Sum, 2015). The actin cytoskeleton is identified as an important component for cellular structure and integrity. Viruses are obligate intracellular parasites that require the involvement of the actin cytoskeleton at all stages, from entry through replication to egress and spread (Taylor et al., 2011). With a few exceptions of viruses that predominantly exploit actin-driven motility, most viruses switch from the actin cytoskeleton to the microtubule tracks to promote long-range movement of their cores (Walsh and Naghavi, 2019). The drug jasplakinolide, which binds to F-actin and results in a decreased rate of actin depolymerization, was previously used to block the turnover of actin microfilaments (Cramer, 1999). Another drug named nocodazole is an antineoplastic agent which exerts its effect in cells by interfering with the polymerization of microtubules (Vasquez et al., 1997). We evaluated the cytotoxic effects of jasplakinolide and nocodazole by the MTT assay, and no apparent cytotoxicity was observed (Figures 6A,D). Treatment with jasplakinolide at concentrations ranging from 0.5 to 2.5 μM resulted in an approximately 80% inhibition of ZIKV infection in T98G cells by qPCR (Figure 6B). We also confirmed that nocodazole blocked ZIKV infection by qPCR (Figure 6E). The production of progeny virions was also reduced upon drug treatment (Figures 6C,F). Treatment of cells with jasplakinolide and nocodazole at the concentrations of 2.5 and 5 μM, respectively, showed a decrease of ZIKV infection in T98G cells by immunofluorescence microscopy (Figures 6G,H). Collectively, these pharmacological and immunofluorescence data strongly suggest that the dynamic reorganization of actin microfilaments and microtubules is critical for the ZIKV infection of T98G cells.

### ZIKV Entry Into T98G Cells Is Independent of Macropinocytosis/Phagocytosis

**FIGURE 7:**
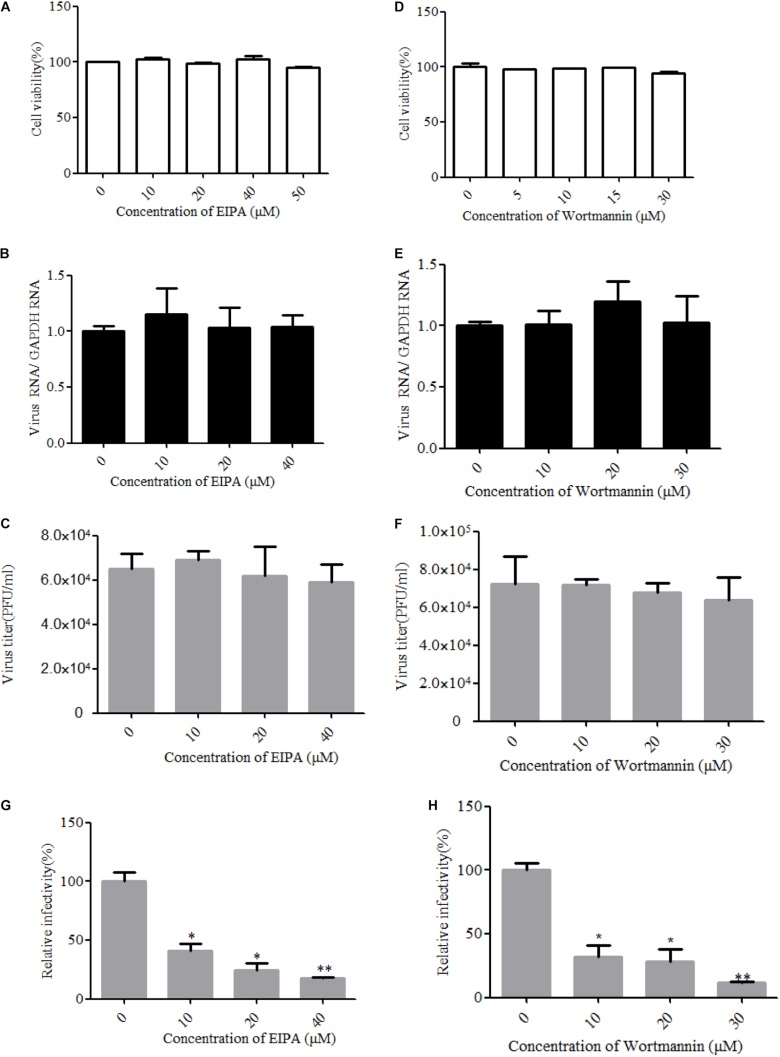
Zika virus (ZIKV) entry is independent of macropinocytosis/phagocytosis. **(A**,**D)** Cell viability upon drug treatment was assessed. **(B**,**C)** T98G cells treated with 5-(*N*-ethyl-*N*-isopropyl)amiloride (EIPA) were incubated with ZIKV at a multiplicity of infection (MOI) of 10 for 1 h at 4°C and then shifted to 37°C for 1 h. The internalized viruses were determined by RT-qPCR **(B)** and infectious center assay **(C)**. **(E**,**F)** T98G cells treated with increasing concentrations of wortmannin were infected with ZIKV at an MOI of 10 for 1 h at 4°C and then shifted to 37°C for 1 h. The internalized viruses were determined by RT-qPCR **(E)** and infectious center assay **(F)**. **(G**,**H)** T98G cells treated with increasing concentrations of EIPA or wortmannin were infected with pseudotyped vesicular stomatitis virus for 1 h at 4°C and then shifted to 37°C for 1 h. The cells were washed by PBS three times and fed with fresh MEM. Luciferase activities were determined at 48 h post-infection. The data shown are the mean ± SD of three independent experiments **(A**–**H)**. *P < 0.05; **P < 0.01.

Macropinocytosis is an actin-driven endocytosis process. Macropinosome formation increases actin polymerization and actin-mediated engulfment on the plasma membrane (Mercer and Helenius, 2009). Macropinocytosis has been reported as an alternative DENV cell entry pathway in HepG2 cells and was found to be important for several viruses (Suksanpaisan et al., 2009). To address the involvement of micropinocytosis in ZIKV entry, we examined the effect of EIPA, an inhibitor of micropinocytosis that blocks Na^+^/H^+^ exchanger activity, thereby modulating Rho GTPase and interfering with macropinosome formation (Yin et al., 2016). Cell viability of the treated cells was unaffected by EIPA at the concentration of 50 μM (Figure 7A). The drug EIPA did not decrease the ZIKV entry into T98G cells (Figures 7B,C). PI3-kinase (PI3K) contributes to a late step in the formation of macropinosomes. We utilized wortmannin, an inhibitor of PI3K, to decrease the macropinosome vesicles (Lee et al., 2005). Cell viability of the treated cells was unaffected by wortmannin at the concentration of 30 μM (Figure 7D). As shown in Figures 7E,F, no inhibition was observed on ZIKV entry after treatment with wortmannin. In contrast, EIPA and wortmannin effectively blocked the internalization of VSVpv (Figures 7G,H). These results together indicate that the ZIKV entry into T98G cells is likely independent of macropinocytosis/phagocytosis.

### Role of Rab Proteins in ZIKV Infection of T98G Cells

**FIGURE 8:**
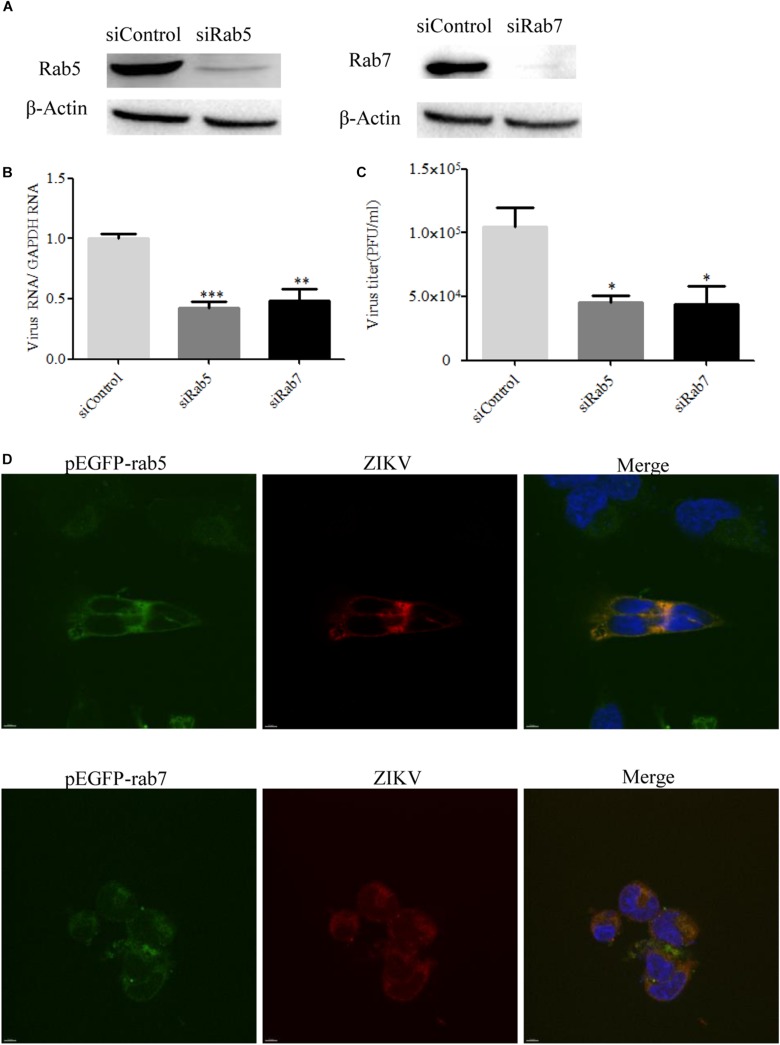
Effects of Rab5 and Rab7 knockdown on Zika virus (ZIKV) infection. **(A)** The knockdown efficiency of Rab5 or Rab7 was determined by Western blotting. T98G cells were transfected with either siControl, siRab5, or siRab7 for 48 h. Cell lysates were reacted with an anti-Rab5 antibody or an anti-Rab7 antibody specific for the indicated proteins. β-actin was used as an internal loading control. One representative experiment out of three is shown. **(B)** Rab5 or Rab7 depletion reduced ZIKV propagation. T98G cells transfected with the indicated siRNAs were infected with ZIKV at a multiplicity of infection (MOI) of 0.5 and incubated for 48 h to allow virus propagation. The infected cells were lysed to quantitate viral RNA copy numbers by RT-qPCR. **(C)** At 48 h post-infection, the titers of supernatant viruses were determined by plaque assay. **(D)** ZIKV localized in Rab5- and Rab7-postitive endosomes. T98G cells were transfected with EGFP-tagged Rab5 or Rab7 for 24 h, followed by infection with ZIKV at an MOI of 10 at 4°C for 1 h and then shifted to 37°C. At 24 h post-infection, the cells were fixed and stained with an anti-ZIKV envelop antibody (*red)*. Nuclei were stained with DAPI (*blue*). One representative experiment out of three is shown **(A)**. The immunofluorescence was examined under a confocal microscope. Representative confocal images from three independent experiments are shown. *Scale bars* in all panels represent 10 μm **(D)**. The data shown are the mean ± SD of three independent experiments **(B**,**C)**. *P < 0.05; **P < 0.01; ***P < 0.001.

It is known that endocytic trafficking is regulated by a large family of small Rab GTPases, with specific Rab GTPases being enriched in distinct intracellular vesicles (Vale-Costa and Amorim, 2016). Rab5 and Rab7 primarily function in early and late endosomes, respectively, and their roles in ZIKV infection remain to be determined. We used Rab5 and Rab7 siRNAs to investigate the requirement for ZIKV transport to early or late endosomes. The knockdown efficiency of siRab5 or siRab7 was confirmed by Western blot. As shown in Figure 8A, the expression level of Rab5 or Rab7 in T98G cells was efficiently knocked down by siRNA treatment. Subsequently, siRNA-transfected cells were infected with ZIKV at an MOI of 0.5. The cells were lysed to measure the copies of viral genomic RNAs by RT-qPCR, while progeny virions were determined by infection assay. The results showed that depletion of Rab5 or Rab7 reduced viral RNA levels compared to those in siControl-transfected cells (Figure 8B). The production of progeny virions was reduced in SiRab (siRab5 and siRab7)-treated cells compared to that in the control group (Figure 8C). In addition, ZIKV appeared to be much less in the Rab5- and Rab7-silenced cells compared to the control cells (Supplementary Figure S3). To determine whether ZIKV localizes in Rab5- or Rab7-postive endosomes, T98G cells were transfected with pEGFP-Rab5 or pEGFP-Rab7. At 24 h post-infection, the cells were incubated with ZIKV at an MOI of 10. The cells were examined by confocal microscopy, and ZIKV was observed in the Rab5- and Rab7-postive endosomes (Figure 8D). The above results together suggest that both Rab5 and Rab7 are likely required for ZIKV endocytosis.

## Discussion

Although there is evidence that ZIKV infects the CNS of the developing fetus (Calvet et al., 2016), the mechanism by which ZIKV causes fetal abnormalities, including microcephaly, remains to be further determined. It has been hypothesized that ZIKV might impair cerebral cortical development by infecting neural progenitor cells (NPCs) (Miner and Diamond, 2016; Tang et al., 2016). Radial glial cells are the primary neural stem cells in the human brain (Fietz et al., 2010; Hansen et al., 2010). Although ZIKV has been shown to infect a range of cells (Khaiboullina S. et al., 2019; Khaiboullina S. F. et al., 2019; Tamhankar and Patterson, 2019), little information is available concerning the infectious cell pathway in glial cells. The glial cell line T98G was derived from human glioblastoma cells in the brain. In this study, we demonstrated that the early infection of ZIKV in T98G cells involves strategies different from those described for other flaviviruses.

Viruses penetrate cells by various mechanisms, including fusion with the cell membrane or entering by receptor-mediated endocytosis (Smith and Helenius, 2004). Clathrin-mediated endocytosis is the most frequently used pathway by many enveloped viruses (Sieczkarski and Whittaker, 2002; van der Schaar et al., 2008). Previously, it was demonstrated that JEV entry into Vero cells is dependent on clathrin-mediated endocytosis and requires low-pH conditions (Nawa et al., 2003). As demonstrated previously, chloroquine inhibited the ZIKV infection of human neural stem cells (Delvecchio et al., 2016). In the current study, the dose-dependent inhibition of the ZIKV entry into T98G cells by NH_4_Cl or chloroquine indicates the involvement of a low pH condition. The pH-dependent entry of ZIKV suggests that the virus is likely to be internalized from the cell surface by receptor-mediated endocytosis and reaches an endosomal compartment where fusion occurs.

Dynamin is a high-molecular-weight GTPase that is required for receptor-mediated endocytosis, including clathrin-mediated and lipid raft/caveola-based endocytosis (Ferguson and De Camilli, 2012). However, little is currently known about the participation of dynamin in the entry process of ZIKV. In this study, the essential role of dynamin in the endocytosis of ZIKV entry was determined. Our data revealed that blockade of dynamin-dependent endocytosis inhibited the ZIKV infection of T98G cells, thus confirming the importance of dynamin.

The information currently available suggests that flavivirus internalization is clathrin-dependent. Our findings firstly showed that a clathrin-dependent endocytosis pathway is operational for the ZIKV entry into T98G cells. Neural cells are highly relevant for ZIKV infection since ZIKV is primarily a neurotropic virus. Previous study by others showed that JEV entry into neural cells is *via* a clathrin-independent pathway (Zhu et al., 2012; Kalia et al., 2013). Our findings demonstrated that the entry of ZIKV into T98G cells is different from that of JEV. In addition, it appears that ZIKV can enter T98G cells through not only clathrin-dependent but also clathrin-independent pathways. Our following findings showed that a caveola-mediated pathway plays an important role in the entry of ZIKV into T98G cells. Of interest is that ZIKV titers decreased 2.5- to 5-fold when the cells were treated with drugs or siRNAs, whereas JEV titers decreased in the range of 5- to 10-fold. This was likely due to the lower infectivity of ZIKV than JEV in T98G cells. We indeed found that ZIKV infection was more efficient in Vero cells than in T98G cells (Supplementary Figure S1).

We also addressed the roles of alternative/minor routes played in ZIKV entry by treatment with specific inhibitors. Cholesterol is required for caveola- and lipid raft-mediated pathway endosomes (Pitha et al., 1988; Rahn et al., 2011). However, cholesterol is essential for most membrane processes, including the endocytosis of viruses (Imelli et al., 2004). Previous studies have shown that cholesterol in cellular membranes is involved during flavivirus infection, suggesting that membrane cholesterol is a requirement for virus infection (Kaufmann and Rossmann, 2011; Zhu et al., 2012; Carro and Damonte, 2013; Yang et al., 2013). We found that the depletion or sequestration of cholesterol from the membrane by MβCD or filipin inhibited the ZIKV entry into T98G cells. These data indicated that lipid raft cholesterol-enriched membrane domains may play an important role in ZIKV infection in T98G cells. In addition, a previous study demonstrated that virus binding induces dynamic rearrangements of the actin cytoskeleton at the early stages of infection (Taylor et al., 2011). Our data from experiments with drugs that alter actin organization also showed that both actin polymerization and depolymerization are required during the ZIKV infection cycles. Macropinocytosis can be defined as a transient, growth factor-induced, actin-dependent endocytic process that leads to the internalization of fluid and membrane in large vacuoles (Mercer et al., 2010). We found that micropinocytosis is unlikely to be involved in the ZIKV entry into T98G cells, as evidenced by EIPA or wortmannin treatment experiments, which is similar to other viruses belonging to the flavivirus family (Zhu et al., 2012; Liu et al., 2017).

Rab GTPases have been shown to be involved in the formation of vesicles on the cell surface and the downstream delivery of internalized molecules to a number of cellular locations (Imelli et al., 2004). Rab5 is a small GTPase associated with the plasma membrane and early endosomes, while Rab7 is critically involved in early to late endosome traffic (Vitelli et al., 1997; Rink et al., 2005). Previous studies with the dengue virus demonstrated that the virus is transported from Rab5-positive early endosomes to Rab7-positive late endosomes before the viral genome is being delivered into the cytoplasm (Krishnan et al., 2007; Acosta et al., 2012). The entry of the West Nile virus requires Rab5, but not Rab7 (Krishnan et al., 2007). In this study, we found that Rab5 and Rab7 are likely required for ZIKV endocytosis. We speculate that ZIKV particles are first transported to the early Rab5-positive endosome, and the late Rab7-positive endosome may provide an important endosomal environment for ZIKV RNA release.

In conclusion, we conduct a systematic study to identify the internalization mechanism of ZIKV into T98G cells. The evidence presented here indicated that ZIKV can enter T98G cells through both clathrin-dependent and clathrin-independent endocytosis pathways.

## Data Availability Statement

The datasets generated for this study are available on request to the corresponding author.

## Author Contributions

QH, ML, and DZ conceived the study. ML and DZ performed the experiments and analyzed the data. CL, MF, FN, YL, and ZZ conducted some experiments. TD, HW, and GG offered advices and technical assistance. ML drafted and QH and MZ reviewed and finalized the manuscript. All authors read and approved the manuscript.

## Conflict of Interest

The authors declare that the research was conducted in the absence of any commercial or financial relationships that could be construed as a potential conflict of interest.
