# Supplementary material for: Characterization of Zika Virus Endocytic Pathways in Human Glioblastoma Cells
Source: Front Microbiol. 2020 Mar 6;11:242. doi: 10.3389/fmicb.2020.00242 (PMC7069030; doi:10.3389/fmicb.2020.00242)
Supplement: Supplementary file 1 [file Data_Sheet_1.pdf]

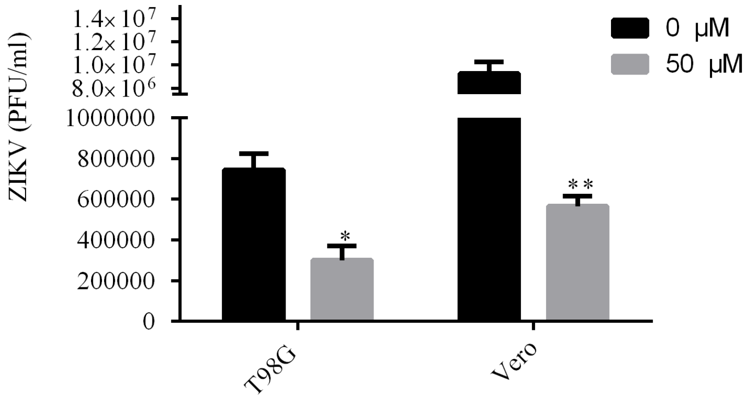

Supplementary Figure 1. ZIKV infection is more efficient in Vero cells than in T98G cells. Following treatment with 50 μM chloroquine, T98G and Vero cells were infected with ZIKV at an MOI of 5. At 48 h post-infection, the titers of supernatant viruses were determined by plaque assay. Data shown are mean ± SD of two independent experiments with each condition performed in triplicate. *P < 0.05; **P < 0.01.

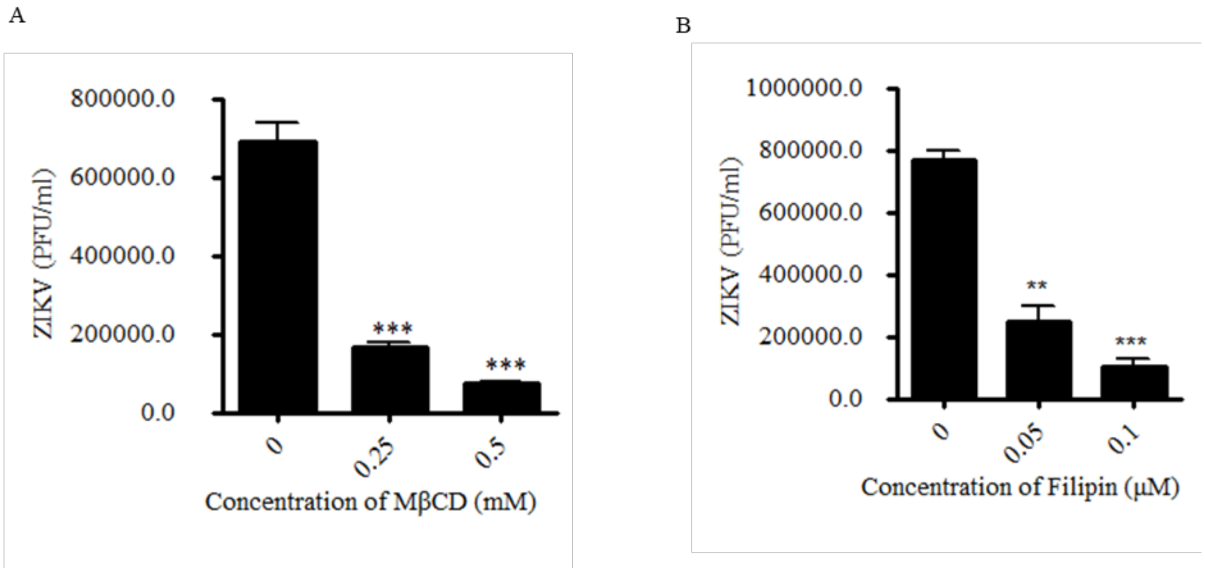

Supplementary Figure 2. MβCD (A) and filipin (B) are virucidal to ZIKV. ZIKV was treated with increasing concentrations of MβCD or filipin for 1 h. The treated ZIKV was chilled, and diluted 100-fold before used for infection. ZIKV infectivity was determined by plaque assay. Data shown are mean ± SD of two independent experiments with each condition performed in triplicate. **P < 0.01; ***P < 0.001.

siControl siRab5 siRab7

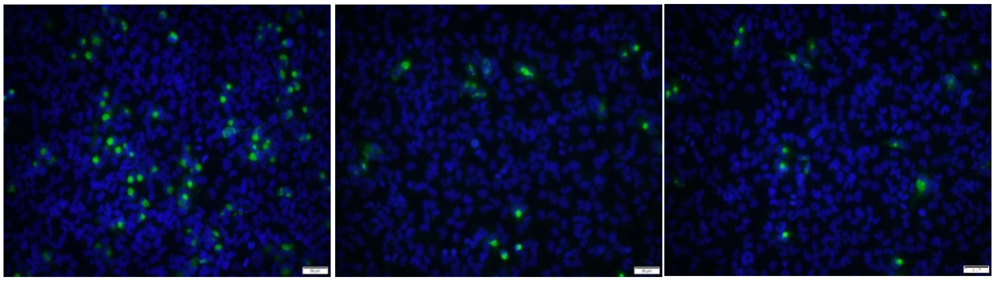

Supplementary Figure 3. ZIKV appears to be much less in Rab5 or Rab7-silenced cells. Cells were transfected with Rab5, Rab7 or control siRNAs for 24 h, and then infected with ZIKV at an MOI of 10. At 1 h post-infection at 37°C, cells were washed with PBS (pH=3.0) to remove unbound viruses on the cell surface. At 24 h post-infection, cells were fixed and stained with an anti-ZIKV envelope antibody (green). Scale bars in all panels represent 50 μm.
